# The Acquisition and Retention of Lumpy Skin Disease Virus by Blood-Feeding Insects Is Influenced by the Source of Virus, the Insect Body Part, and the Time since Feeding

**DOI:** 10.1128/jvi.00751-22

**Published:** 2022-07-12

**Authors:** Beatriz Sanz-Bernardo, Rey Suckoo, Ismar R. Haga, Najith Wijesiriwardana, Alice Harvey, Sanjay Basu, Will Larner, Sara Rooney, Victoria Sy, Zoë Langlands, Eric Denison, Christopher Sanders, John Atkinson, Carrie Batten, Luke Alphey, Karin E. Darpel, Simon Gubbins, Philippa M. Beard

**Affiliations:** a The Pirbright Institute, Pirbright, Surrey, United Kingdom; b MSD Animal Health, Milton Keynes, United Kingdom; University of California, Irvine

**Keywords:** poxvirus, lumpy skin disease, transmission, mosquitoes, flies, midges, vector, control, *Aedes aegypti*, *Culex quinquefasciatus*, *Stomoxys calcitrans*, *Culicoides nubeculosus*

## Abstract

Lumpy skin disease virus (LSDV) is a poxvirus that causes severe systemic disease in cattle and is spread by mechanical arthropod-borne transmission. This study quantified the acquisition and retention of LSDV by four species of Diptera (Stomoxys calcitrans, Aedes aegypti, Culex quinquefasciatus, and Culicoides nubeculosus) from cutaneous lesions, normal skin, and blood from a clinically affected animal. The acquisition and retention of LSDV by Ae. aegypti from an artificial membrane feeding system was also examined. Mathematical models of the data were generated to identify the parameters which influence insect acquisition and retention of LSDV. For all four insect species, the probability of acquiring LSDV was substantially greater when feeding on a lesion compared with feeding on normal skin or blood from a clinically affected animal. After feeding on a skin lesion LSDV was retained on the proboscis for a similar length of time (around 9 days) for all four species and for a shorter time in the rest of the body, ranging from 2.2 to 6.4 days. Acquisition and retention of LSDV by *Ae. aegypti* after feeding on an artificial membrane feeding system that contained a high titer of LSDV was comparable to feeding on a skin lesion on a clinically affected animal, supporting the use of this laboratory model as a replacement for some animal studies. This work reveals that the cutaneous lesions of LSD provide the high-titer source required for acquisition of the virus by insects, thereby enabling the mechanical vector-borne transmission.

**IMPORTANCE** Lumpy skin disease virus (LSDV) is a high consequence pathogen of cattle that is rapidly expanding its geographical boundaries into new regions such as Europe and Asia. This expansion is promoted by the mechanical transmission of the virus via hematogenous arthropods. This study quantifies the acquisition and retention of LSDV by four species of blood-feeding insects and reveals that the cutaneous lesions of LSD provide the high titer virus source necessary for virus acquisition by the insects. An artificial membrane feeding system containing a high titer of LSDV was shown to be comparable to a skin lesion on a clinically affected animal when used as a virus source. This promotes the use of these laboratory-based systems as replacements for some animal studies. Overall, this work advances our understanding of the mechanical vector-borne transmission of LSDV and provides evidence to support the design of more effective disease control programmes.

## INTRODUCTION

Lumpy skin disease virus (LSDV) is a poxvirus that causes severe, systemic disease in cattle and water buffalo. Over the past 10 years, LSDV has spread into the Middle East, Europe, and Asia, resulting in substantial production and economic losses to the cattle industry in these regions. An incomplete understanding of the transmission of LSDV has hampered the design of effective and proportionate interventions, making the epidemic difficult to control.

Poxviruses, including LSDV, have previously been shown to be transmitted via hematophagous (blood-feeding) arthropod vectors. Evidence to date suggests that poxviruses use a mechanical form of vector-borne transmission, without any multiplication of the virus in the vector. This is in contrast to biological vector-borne transmission, which incorporates a replication stage of the pathogen within the vector. Mechanically transmitted viruses are characterized by a low specificity for the transmitting vector, and they require a high titer virus source to enable acquisition (1). Examples of mechanically transmitted viruses include equine infectious anemia virus (2), bovine leukemia virus (3), and the poxviruses fowlpox virus (4), Shope fibroma virus (5), myxoma virus (6), and LSDV (7).

Epidemiological data from LSD outbreaks are consistent with LSDV transmission by arthropod vectors (8–13). These observations are supported by experimental work which has shown that LSDV can be transmitted from clinical donor to naive recipient via Aedes aegypti (7), *Haematopota* spp. horse flies (14), and the biting flies Stomoxys calcitrans, Stomoxys sitiens, and Stomoxys indica (14, 15). Experimental transmission of LSDV (but not the disease) has also been demonstrated via Rhipicephalus appendiculatus ticks (16). The literature also suggests that direct contact (transmission from one animal to another without vector involvement) is an uncommon mechanism of LSDV transmission (17, 18). A better understanding of the nature of vector-borne transmission of LSDV will improve knowledge of LSD epidemiology and potentially provide new intervention targets for LSD control and prevention programs.

Our recent work on the acquisition and retention of LSDV in hematophagous insects found broad vector specificity for LSDV acquisition from clinically affected cattle, and no evidence of viral replication in the vector (19), consistent with a mechanical mode of transmission. In this study, we investigate the vector-borne transmission of LSDV in more detail. We quantify the acquisition of the virus and its retention in different anatomical locations of four vector species (*S. calcitrans*, *Ae. aegypti*, Culex quinquefasciatus, and Culicoides nubeculosus). We discover that LSDV is acquired principally from skin nodules of clinically affected cattle and remains for longer on the mouthparts of the insect (around 9 days) compared to the body (2.2 to 6.4 days). We also develop and utilize artificial membrane feeding systems for the acquisition and retention of LSDV by insect species to facilitate study of the factors which influence these elements of viral transmission.

## RESULTS

**FIG 1:**
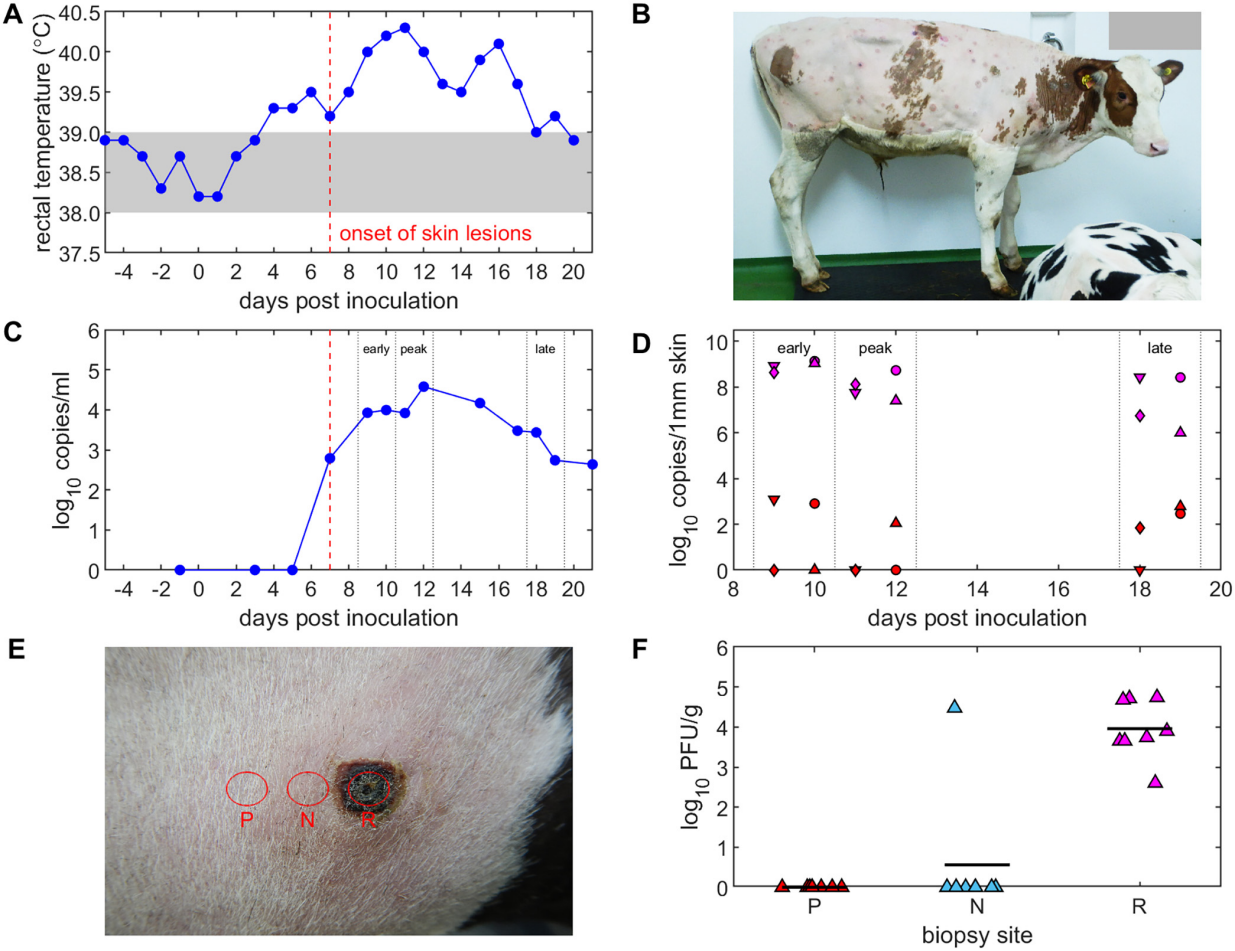
Clinical signs and levels of lumpy skin disease (LSD) viral DNA and virus in the donor calf. (A) Rectal temperature (°C). (B) Gross pathology of experimental LSD in the donor calf. (C) Levels of viral DNA in blood (log_10_ copies/mL). (D) Levels of viral DNA (log_10_ copies/1 mm skin) in lesions (magenta) and normal skin (red) to which different insects were exposed: Aedes aegypti (circles); Culex quinquefasciatus (up-triangles); Culicoides nubeculosus (down-triangles); and Stomoxys calcitrans (diamonds). (E) Example of a lesion on the calf and areas where samples were taken (red circles). (F) Levels of infectious virus (log_10_ PFU/g) in samples taken from the center of a lesion (R), adjacent to a lesion (N), or approximately 10 mm from the lesion (P).

Four calves were challenged with LSDV intradermally and intravenously, as reported previously (20). One calf developed clinical signs consistent with LSD, including fever (Fig. 1A) and multiple cutaneous nodules (Fig. 1B). The amount of virus present in the blood of the clinical calf was calculated to be between 5.5 × 10^2^ and 3.8 × 10^4^ copy numbers/mL blood (Fig. 1C) and a maximum of 30 PFU/mL (detected only at 9 and 11 days postinoculation [dpi]). A large amount of viral DNA was present in the microbiopsy samples taken from skin nodules, between 9.8 × 10^5^ and 1.3 × 10^9^ copy numbers per 1-mm diameter skin biopsy sample. In contrast, the amount of viral DNA detected in microbiopsy samples taken from “normal skin” feeding sites was much lower, up to a maximum of 1.2 × 10^3^ copy numbers per 1-mm diameter skin biopsy sample but undetectable in 6 of the 12 samples of normal skin (Fig. 1D).

We investigated further this difference in the amount of virus present in lesioned and normal skin. Titration of infectious virus could not be carried out on microbiopsy samples due to the small amount of tissue present; therefore, we collected biopsy samples (8-mm diameter) from the skin of another clinical calf during postmortem examination (21 dpi). This calf was part of a subsequent experiment conducted at The Pirbright Institute, and was housed and inoculated in the same manner as described above. Biopsy samples were taken from a lesion, from the raised area just adjacent to a lesion, or from normal skin approximately 10 mm from a lesion (Fig. 1E). This was repeated for a total of 8 lesions. Virus was detected in all eight biopsy samples taken from the centers of the lesions (1 × 10^3^ to 1.6 × 10^5^ PFU/g tissue), one of the eight samples from the raised area just adjacent to the lesion (1 × 10^5^ PFU/g), and none of the samples of skin approximately 10 mm from the lesion (limit of detection: 50 to 100 PFU/g tissue) (Fig. 1F). This indicates that at 21 dpi, LSDV is highly concentrated in the cutaneous lesions of clinically affected cattle, while the tissue immediately surrounding the lesion has little to no virus.

The acquisition of LSDV by hematogenous arthropods from the clinically affected calf in Fig. 1B was studied in detail. A total of 441 insects were fed on either a lesion, normal skin, or an artificial membrane system (Hemotek, United Kingdom) containing viremic blood collected from the clinically affected calf (Table 1). Insects which had fed were incubated for 0, 2, 4, or 8 days, then dissected into proboscis, head-thorax (including the upper digestive tract and salivary glands), and abdomen (*C. nubeculosus*, *Ae. aegypti*, and *Cx. quinquefasciatus*) or proboscis and head-thorax-abdomen (*S. calcitrans*).

**TABLE 1:** Number of insects of each species tested for lumpy skin disease viral DNA at each time point post feeding

Species	Normal skin				Lesion				Hemotek			
	0 dpf^*a*^	2 dpf	4 dpf	8 dpf	0 dpf	2 dpf	4 dpf	8 dpf	0 dpf	2 dpf	4 dpf	8 dpf
Aedes aegypti	12	12	11	12	11	12	12	12	8	7	8	8
Culex quinquefasciatus ^*b*^	16 (12)	7 (4)	8 (4)	8 (4)	8 (4)	8 (4)	7 (4)	8 (4)	8	8	8	8
Culicoides nubeculosus	12	12	8	15	11	12	8	16	8	8	8	8
Stomoxys calcitrans	11	11	9	4	12	11	7	3	7	8	3	2

a dpf: days post-feeding.

b Data on the level of viral DNA in normal skin or lesions was not available for *Cx. quinqefasciatus* fed at 20 days post inoculation; the number in brackets is the number included in the analysis.

**FIG 2:**
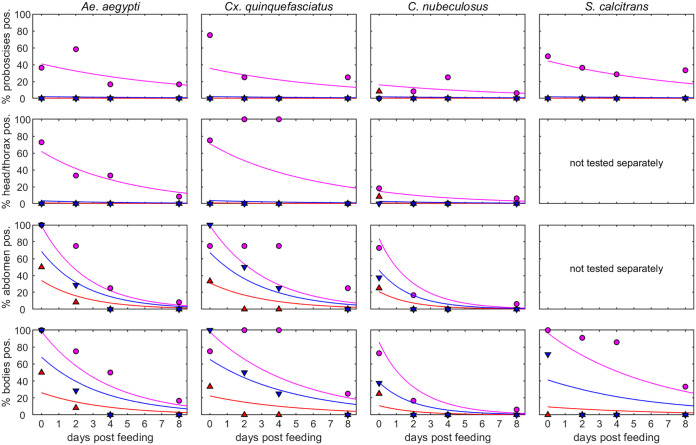
The proportion of insect parts positive for lumpy skin disease viral DNA depends on virus source, body part tested, and time post-feeding for four species of biting insect. Each plot shows the observed proportion of positive insects (symbols) and the posterior median for the expected proportion of positive insects (lines). Virus source is indicated by color and symbol: normal skin of a clinical calf (red up-triangles), a lesion on a clinical calf (magenta circles), or blood from a clinical calf via an artificial membrane feeding system (blue down-triangles). The body part tested (proboscis, head/thorax, abdomen, or body) is indicated in the *y* axis label.

LSDV genomic DNA was detected in all three sections of *Ae. aegypti* (the proboscis, head/thorax, and abdomen) at 0, 2, 4, and 8 days post-feeding (dpf) on a lesion (Fig. 2). However, LSDV DNA was not present in any proboscis or head/thorax portions of *Ae. aegypti* which had fed on normal skin or the artificial membrane system and was only detectable in the abdominal portion up to 2 dpf. A similar trend was seen in *Cx. quinquefasciatus* (Fig. 2), with multiple portions of proboscis and head virus-positive after feeding on a skin lesion, but very little virus detected in the proboscis or head of insects fed on normal skin or the artificial membrane system.

Fewer *C. nubeculosus* were positive for LSDV DNA compared to the two mosquito species (Fig. 2), with most virus-positive body portions found in individuals which had fed on skin lesions. All 12 *S. calcitrans* flies which were exposed to skin lesion were positive for viral DNA in the head-thorax-abdomen portion on day 0, and 6 (out of 12) also had viral DNA detectable in the proboscis portion, indicating very efficient acquisition of the virus from the lesion (Fig. 2). Viral DNA was still detectable in both portions at 8 dpf, demonstrating long-term retention. In contrast, no virus-positive flies were detected after being exposed to normal skin, and flies that had fed on the artificial membrane system were positive only on day 0 and only in the head-thorax-abdomen portion. This indicates that feeding on a lesion is required for LSDV to become associated with the proboscis or head/thorax of the insect, and also required for long-term (greater than 2 days) retention of the virus.

**FIG 3:**
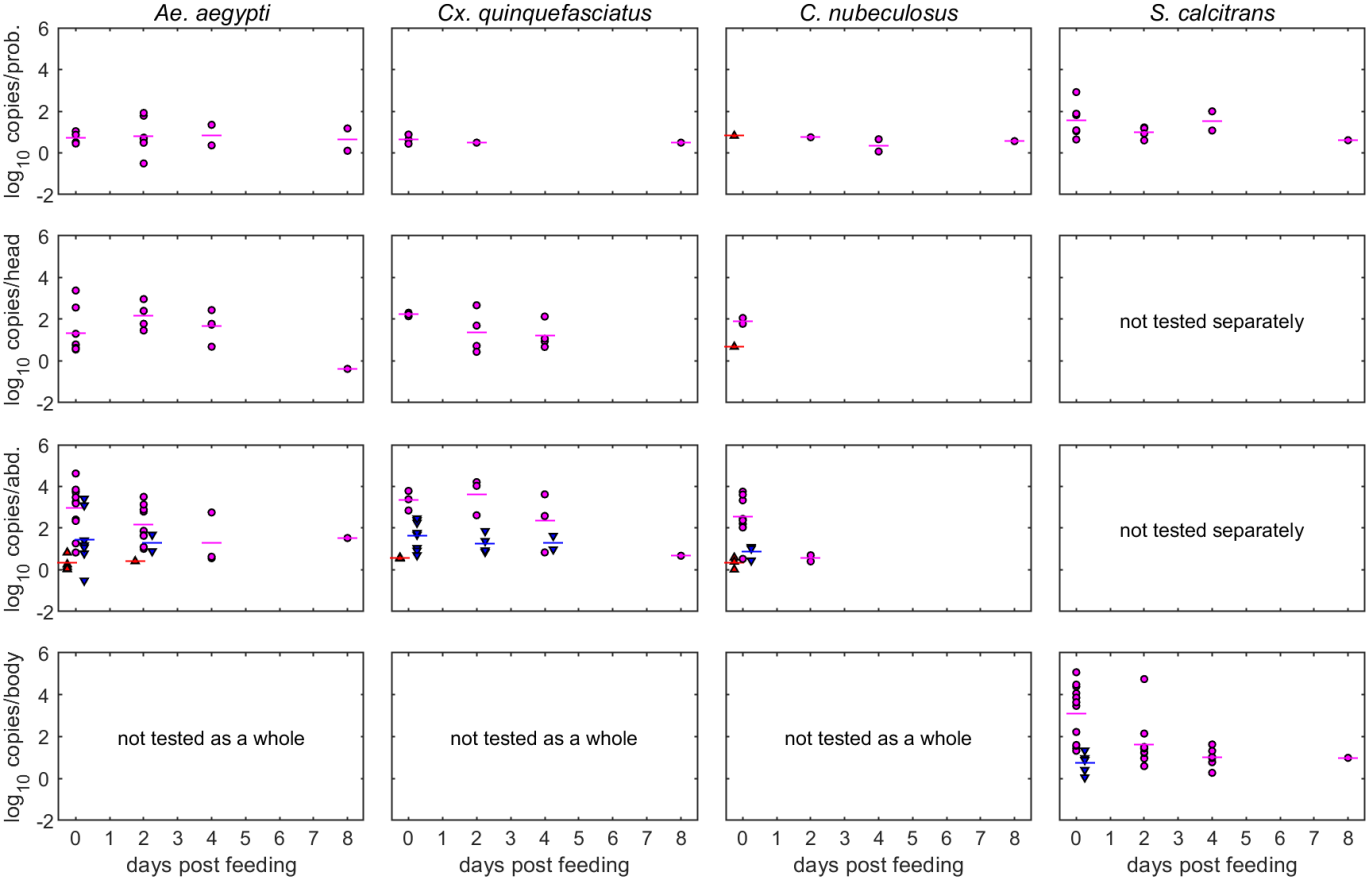
Levels of retained lumpy skin viral DNA depends on virus source, body part tested, and time post-feeding for four species of biting insect. Each plot shows the level of viral DNA retained in each part (log_10_ copy number/part; symbols) and the mean level retained (horizontal lines). Virus source is indicated by color and symbol: normal skin of a clinical calf (red up-triangles), a lesion on a clinical calf (magenta circles), or blood from a clinical calf via an artifical membrane feeding system (blue down-triangles). The body part tested (proboscis [prob.], head/thorax [head], abdomen [abd.], or body) is indicated in the *y* axis label.

In addition to analyzing the number of insect portions which were virus-positive at each time point, the amount of viral DNA present in the portions was also studied (Fig. 3). The amount of LSDV genomic DNA present in or on the proboscises of the four insect species was low (rarely more than 100 genome copies per proboscis) but consistent over the 8 days post-feeding. In contrast, the amount of LSDV genomic DNA in the abdomen (*Ae. aegypti*, *Cx. quinquefasciatus*, and *C. nubeculosus*) and body portions (*S. calcitrans*) decreased over time.

Mathematical models were then generated to enable a more detailed study of the parameters which influence the acquisition and retention of LSDV in insects. Models were generated to cover the two different dissection strategies (two portions [analysis 1] or three portions [analysis 2]). For both analyses, comparison of different models indicated that the best-fitting are those in which the probability of acquisition depends on both virus source and insect part, and the probability of retention depends on insect part (see Appendix). Furthermore, the effects of both virus source and insect part vary among species (see Appendix). The final models adequately capture the data with the observed proportion of virus-positive insects lying within the 95% posterior predictive intervals for the models (not shown).

The probability of acquiring LSDV was substantially greater when feeding on a lesion compared with feeding on normal skin for all four species (odds ratios [OR]: 200 [*Ae. aegypti*]; 164 [*Cx. quinquefasciatus*]; 55 [*C. nubeculosus*]; 299 [*S. calcitrans*], Table 2). The probability of acquiring LSDV was also greater when feeding on blood from a clinical calf via a Hemotek compared with feeding on normal skin, with the odds ratio similar for all four species (OR = 6) (Table 2). Furthermore, the body of an insect was more likely to be positive immediately after feeding than the proboscis (OR = 99 for *Ae. aegypti* and *Cx. quinquefasciatus*; OR = 33 for *C. nubeculosus* and *S. calcitrans*) (Table 2).

**TABLE 2:** Parameter estimates for the effects of virus source and insect body part on the probability of acquisition and retention of lumpy skin disease virus by four species of biting insect

parameter^*a*^	*Ae. aegypti*		*Cx. quinquefasciatus*		*C. nubeculosus*		*S. calcitrans*
	Analysis 1	Analysis 2	Analysis 1	Analysis 2	Analysis 1	Analysis 2	Analysis 1
*probability of acquisition*							
intercept	−5.7 (−7.2, −4.4)	−5.4 (−7.0, −4.2)	−5.8 (−7.2, −4.5)	−5.5 (−7.0, −4.3)	−5.6 (−7.1, −4.3)	−5.4 (−6.8, −4.2)	−5.4 (−7.8, −4.6)
insect part							
proboscis	0 (baseline)	0 (baseline)	0 (baseline)	0 (baseline)	0 (baseline)	0 (baseline)	0 (baseline)
head/thorax	–	0.6 (−0.4, 4.6)	–^*c*^	0.6 (−0.4, 1.7)	–	0.6 (−0.5, 1.6)	–
abdomen	–	4.8 (3.5, 6.5)	–	4.7 (3.4, 6.2)	–	4.0 (2.5, 5.4)	–
body	4.6 (3.3, 6.5)	–	4.5 (3.2, 6.1)	–	3.5 (2.1, 4.9)	–	3.6 (2.4, 4.9)
virus source^*b*^							
normal skin	0 (baseline)	0 (baseline)	0 (baseline)	0 (baseline)	0 (baseline)	0 (baseline)	0 (baseline)
iesion	5.3 (4.0, 7.0)	5.3 (4.1, 6.8)	5.1 (3.7, 6.9)	5.7 (4.3, 7.5)	4.0 (2.4, 5.7)	3.1 (1.8, 4.4)	5.7 (4.3, 7.7)
viremic blood	1.8 (0.8, 3.0)	1.4 (0.5, 2.6)	1.9 (0.9, 3.1)	1.5 (0.6, 2.7)	1.7 (0.4, 2.8)	1.2 (−0.1, 2.3)	1.9 (0.9, 3.6)
dose-response^*b*^	0.9 (0.7, 1.1)	0.9 (0.7, 1.2)	1.0 (0.7, 1.3)	1.1 (0.9, 1.4)	0.6 (0.4, 0.9)	0.6 (0.4, 0.8)	1.0 (0.8, 1.2)
*probability of retention*							
decay rate (/day)							
proboscis	0.1 (0.02, 0.2)	0.1 (0.02, 0.3)	0.1 (0.02, 0.3)	0.1 (0.02, 0.3)	0.1 (0.01, 0.3)	0.1 (0.02, 0.3)	0.1 (0.01, 0.2)
head/thorax	–	0.2 (0.08, 0.3)	–	0.2 (0.05, 0.3)	–	0.2 (0.06, 0.4)	–
abdomen	–	0.4 (0.3. 0.5)	–	0.3 (0.2, 0.4)	–	0.5 (0.3, 0.8)	–
body	0.3 (0.2, 0.4)	–	0.2 (0.1, 0.3)	–	0.5 (0.3, 0.8)	–	0.2 (0.06, 0.3)
mean duration of retention (days)							
proboscis	8.9 (4.3, 64.8)	7.3 (3.6, 45.1)	8.7 (3.8, 64.9)	6.8 (3.1, 45.3)	8.9 (3.8, 75.1)	7.9 (3.6, 59.7)	9.1 (1.4, 79.7)
head/thorax	–	5.2 (3.0, 13.1)	–	6.4 (3.3, 18.7)	–	5.4 (2.7, 16.7)	–
abdomen	–	2.7 (1.9, 3.8)	–	3.2 (2.3, 5.7)	–	2.1 (1.3, 3.1)	–
body	3.8 (2.7, 5.7)	–	5.1 (3.2, 10.6)	–	2.2 (1.2, 3.9)	–	6.4 (3.5, 17.6)

a Posterior median (95% credible interval).

b Virus source is included in the model given by equation 1 and dose-response is included in the model given by equation 2.

c Dashes mean that the analysis was not performed.

A similar effect of virus source on the probability of acquisition was seen in analysis 2 (Table 2). This analysis also indicated that the head/thorax of an insect was more likely than its proboscis to be positive immediately after feeding (OR = 1.8 for all three species), though this increase was not significant (Table 2). In addition, the abdomen of an insect was considerably more likely to be positive than the proboscis (OR = 122 [*Ae. aegypti*]; 110 [*Cx. quinquefasciatus*]; 33 [*C. nubeculosus*]) or the head/thorax (OR = 66 [*Ae. aegypti*]; 55 [*Cx. quinquefasciatus*]; 30 [*C. nubeculosus*]) (Table 2).

LSDV was retained on the proboscis for a similar length of time (around 9 days) for all four species and for a longer time than in the body (Fig. 2; Table 2). The mean duration of LSDV retention in the body differed among species and ranged from 2.2 days for *C. nubeculosus* to 6.4 days for *S. calcitrans* (Table 2). In analysis 2, LSDV was retained longest on the proboscis (mean duration: 6.4 to 7.9 days), followed by the head/thorax (5.2 to 6.4 days), and for the shortest time in the abdomen (2.1 to 3.3 days).

**FIG 4:**
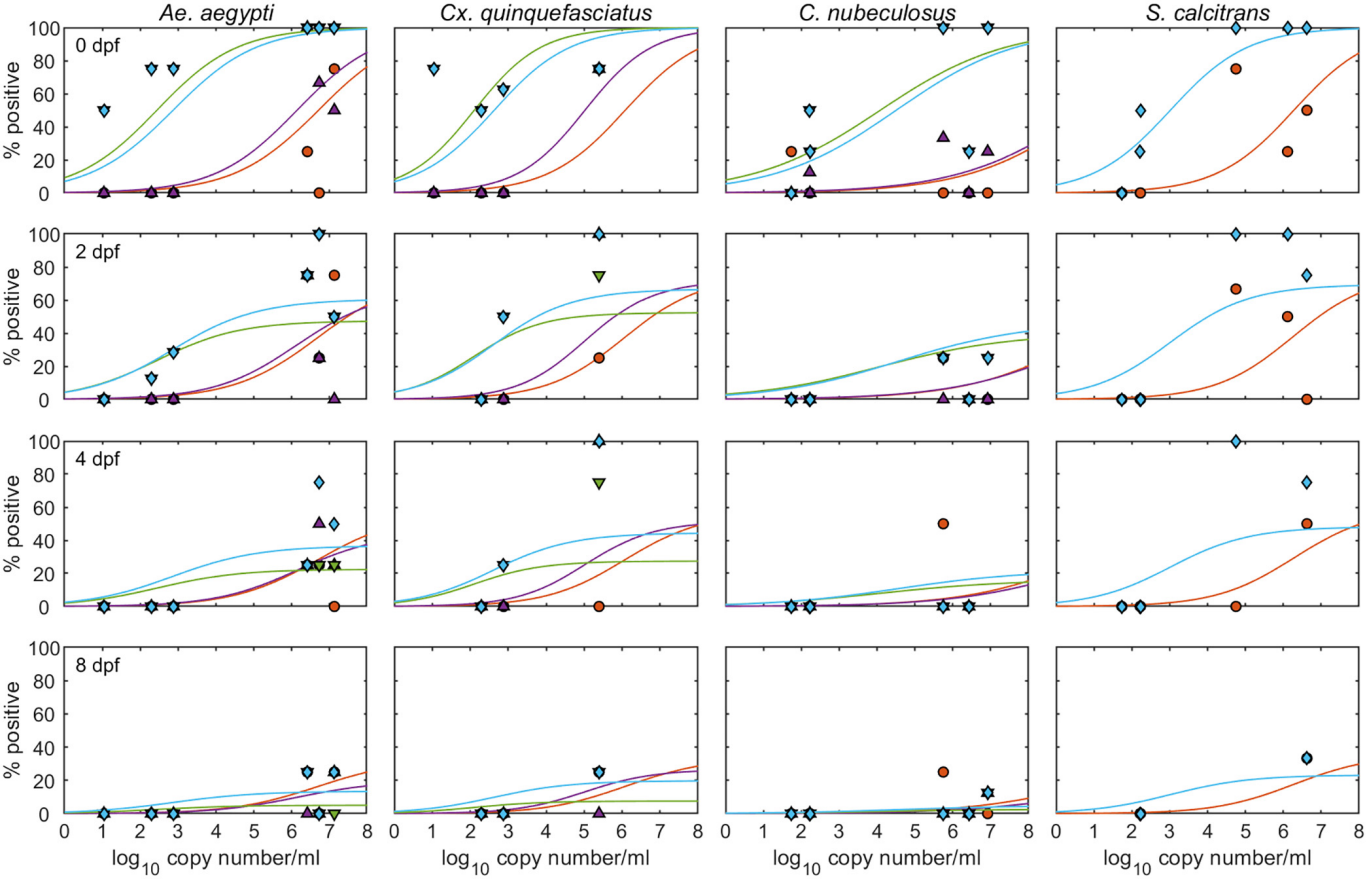
Relationship between level of lumpy skin disease viral DNA and the probability of virus acquisition. Each plot shows the dose-response relationship between the probability of an insect part (proboscis, head/thorax, abdomen, or body) being positive and the level of viral DNA to which the insect was exposed at feeding (log_10_ copy number/mL). Four species of insect (indicated at the top of each column) were tested at 0, 2, 4, and 8 days post-feeding (dpf, rows). Plots show the observed proportion of positive proboscises (orange circles), head/thoraxes (purple up-triangles), abdomens (green down-triangles), and bodies (blue diamonds) and the posterior median probability of an insect part being positive (lines: proboscis, orange; head/thorax, purple; abdomen, green; body, blue).

The differences between sources of virus in the probability of acquiring LSDV primarily reflects differences in the levels of viral DNA to which an insect is exposed. In both analyses, this was demonstrated by fitting the model, including the probability of acquisition given by equation 2, to the data (Fig. 4; Table 2), which yielded an equally good fit to the data as one in which the probability of acquisition was given by equation 1 (based on posterior predictive *P* values). Differences between insect parts in the probabilities of acquisition and retention were similar in the two models. The dose-response parameters were the same for each species in analyses 1 and 2 (Table 2; cf. Fig. 4).

Once the acquisition and retention of LSDV by insects exposed to a clinical animal had been characterized, we next developed laboratory models of an LSD cutaneous lesion and compared the acquisition and retention of LSDV by *Ae. aegypti*. The *ex vivo* skin laboratory model incorporated thin layers of a LSDV cutaneous nodule from a calf with clinical LSD. This tissue was layered between parafilm membrane and placed over a blood meal in an artificial membrane system. Alongside the *ex vivo* skin model, we also tested two *in vitro* models, consisting of a blood meal spiked with LSDV to a titer similar to the viremia detected during natural LSD (approximately 5 × 10^3^ copy numbers/mL) and one spiked with LSDV to a titer similar to the viral load observed in skin lesions during natural LSD (approximately 4 × 10^7^ copy numbers/mL).

The data from the insects which had fed on the three laboratory models was analyzed using mathematical modeling in order to compare the probabilities of acquisition and retention of LSDV. The probability of acquisition depended on the source of the virus and insect part, while the probability of retention depended on insect part (see Appendix). The final mathematical model adequately captures the data, with the observed proportion of positive insects lying within the 95% posterior predictive intervals for the models (data not shown).

**FIG 5:**
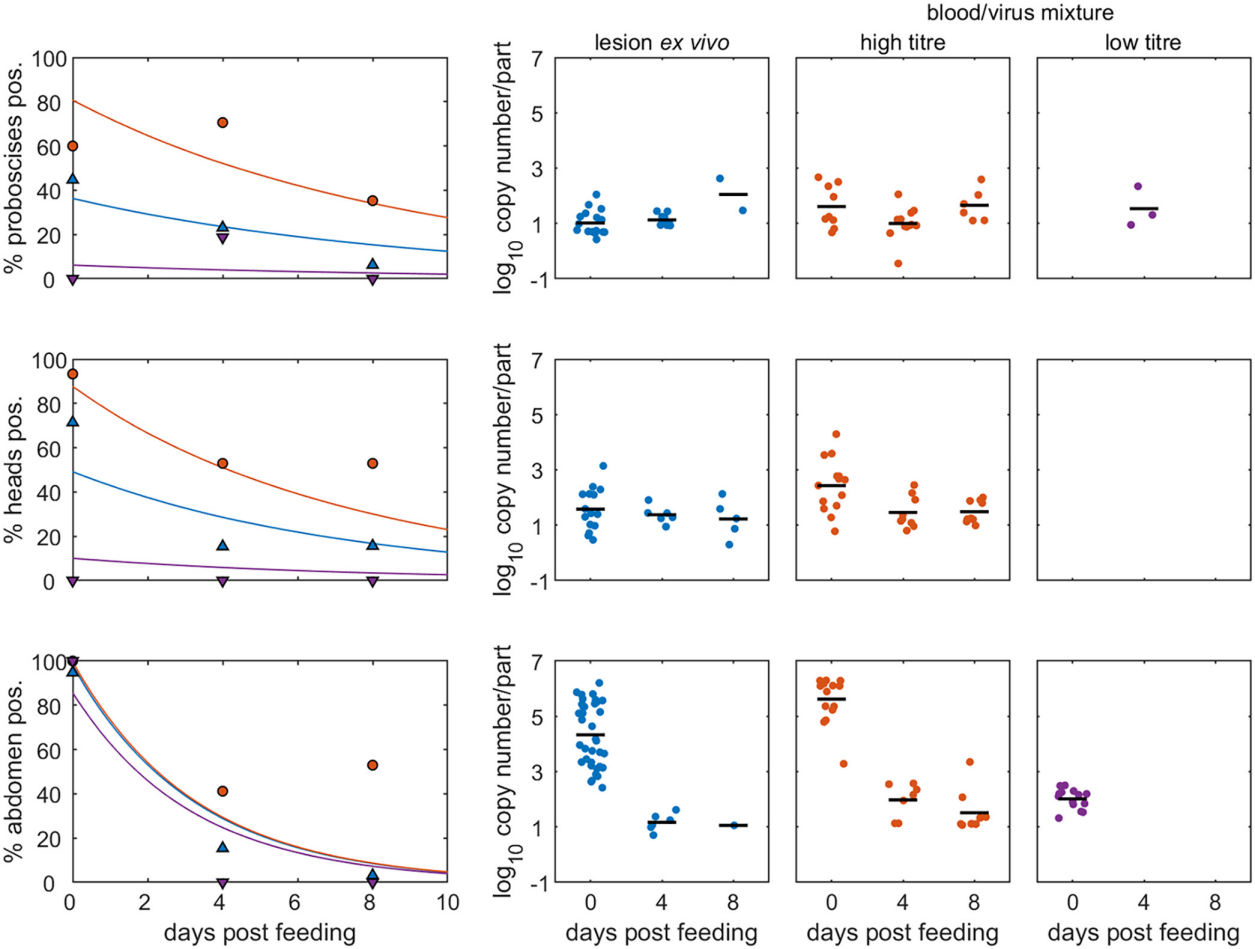
Acquisition and retention of lumpy skin viral DNA in different body parts of Aedes aegypti after feeding *ex vivo*: proboscis (top row), head/thorax (middle row), or abdomen (bottom row). The first column shows the proportion of insect parts positive for viral DNA. Each plot shows the observed proportion of positive parts (symbols) and the posterior median for the expected proportion of positive parts (lines). The second, third, and fourth columns show the levels of lumpy skin viral DNA (log_10_ copy number/part) retained in each part. The circles in each plot show the levels for individual parts and the solid black line indicates the mean. In each panel, the source of virus is indicated by color: lesion *ex vivo* (blue); blood/virus mixture, high titer (orange); or blood/virus mixture, low titer (purple).

Acquisition and retention of LSDV by *Ae aegypti* from the *ex vivo* skin-layering laboratory model was very effective with LSDV, detected at 0, 4, and 8 dpf in the proboscis, head/thorax, and abdomen (Fig. 5). LSDV was also detected in all three portions of *Ae aegypti* at 0, 4, and 8 d after feeding on the high-viremia *in vitro* laboratory model. However, virus acquisition and retention were lower in insects which had fed on the low viremia *in vitro* laboratory model, with very few virus-positive proboscis or head/thorax portions and no virus-positive portions at 8 dpf.

The level of retained viral DNA in *Ae. aegypti* varied among insect parts, virus source, and days post-feeding. The level of viral DNA was highest in the abdomen of insects feeding on blood/virus mix at high titer, followed by the abdomen of those feeding on a lesion *ex vivo* (Fig. 5). The level of viral DNA in the abdomen declined significantly with time since feeding, regardless of virus source. In contrast, there were no significant (*P* > 0.05) differences in levels of viral DNA on the proboscis or head/thorax for either virus source or at different days post-feeding.

An insect feeding on the high-titer *in vitro* laboratory model was considerably more likely to be positive for viral DNA than one feeding on a lesion *ex vivo* (OR = 7) or feeding on a low-titer *in vitro* laboratory model (OR = 64) (Table 3). Differences in the probability of acquisition among insect parts were similar after feeding on the laboratory models and after feeding on a calf (Table 3; cf. Table 2). In addition, LSDV DNA was retained in/on the proboscis, head/thorax, and abdomen after feeding on the laboratory models and after feeding on a calf (Table 3; cf. Table 2).

**TABLE 3:** Parameters describing the effect of virus source and insect body part on the probability of acquisition and retention of lumpy skin disease virus by Aedes aegypti

Parameter^*a*^	Estimate	
	*Ex vivo* study only^*b*^	*Ex vivo* and animal studies
*probability of acquisition*		
intercept	−0.6 (−1.1, −0.04)	−0.5 (−1.0, −0.05)
virus source		
lesion *ex vivo*	0 (baseline)	0 (baseline)
blood/virus mix, high titre	2.0 (1.2, 2.9)	2.0 (1.2, 2.9)
blood/virus mix, low titre	−2.2 (−3.3, −1.2)	−2.4 (−3.6, −1.4)
normal skin on a clinical calf	–	−5.8 (−8.1, −4.1)
lesion on a clinical calf	–	0.4 (−0.3, 1.1)
viremic blood from a clinical calf	–	−3.7 (−5.8, −2.3)
insect part		
proboscis	0 (baseline)	0 (baseline)
head/thorax	0.5 (−0.3, 1.4)	0.8 (−0.1, 1.3)
abdomen	4.5 (3.1, 6.6)	5.7 (4.2, 7.8)
*probability of retention*		
decay rate		
proboscis	0.1 (0.05, 0.2)	0.1 (0.06, 0.2)
head/thorax	0.1 (0.07, 0.2)	0.2 (0.09, 0.2)
abdomen	0.3 (0.2, 0.4)	0.3 (0.3, 0.4)
mean duration of retention (days)		
proboscis	9.4 (5.8, 20.2)	9.0 (5.8, 17.4)
head/thorax	7.5 (4.9, 14.6)	6.6 (4.7, 11.2)
abdomen	3.3 (2.6, 4.1)	3.0 (2.5, 3.7)

a Posterior median (95% credible interval).

b Dashes means the estimate was not calculated.

Comparisons of the acquisition and retention of LSDV by *Ae. aegypti* from all six feeding mechanisms showed that the probability differed significantly among virus sources (Table 3). The probability of acquiring viral DNA from a lesion on a calf was slightly but not significantly higher (OR = 1.47) than that when feeding on a lesion *ex vivo* (Table 3). When feeding on viremic blood via an artificial membrane system, an insect was significantly less likely to acquire viral DNA than when feeding on a spiked blood/virus mix at both low (OR = 0.26) and high (OR = 0.003) titers via an artificial membrane system (Table 3).

## DISCUSSION

This research compared the acquisition and retention of LSDV by four species of insects after feeding on *in vivo*, *ex vivo*, and *in vitro* sources of the virus. Initially we examined insects that had fed on a calf with systemic LSD including numerous skin lesions. LSDV acquisition was far greater for all four insect species when fed on skin lesions compared to that of insects that had fed on nonaffected, normal areas of skin on the calf. This difference was consistent with the far greater numbers of infectious viral units found in skin lesions compared to normal skin. We found that LSDV was tightly concentrated in skin lesions (up to 1.6 × 10^5^ PFU/g) but low to undetectable in samples of “normal” skin, including areas adjacent to lesions. The importance of the skin lesions for acquisition of LSDV by insects strengthens the view that the cohort of animals in a herd which develop cutaneous lesions during an outbreak (approximately 10% of the herd) are those which drive the transmission of LSD and should be the focus of control measures. In comparison, cattle without skin lesions are likely to be much less important in LSDV transmission. Assuming that the probability of acquiring LSDV from a nonclinical animal is the same as that for feeding on viremia blood, an insect feeding on a nonclinical animal is around 79% less likely to acquire LSDV than one feeding on lesions on a clinical one (Table 2). This level of reduction is the same as what we observed in previous transmission experiments (19). This lower probability of acquisition results in a basic reproductive number for transmission via nonclinical cattle of below one for all species except *S. calcitrans* (19).

To study the acquisition and retention of LSDV in more detail, we examined the location of LSDV in insects post-feeding. LSDV was found in the abdominal portion immediately after feeding on a source of virus, consistent with entry to the midgut. The quantity of LSDV in the abdominal portion reduced rapidly over time, consistent with excretion of virus (21) or degradation by the digestive process. In contrast, LSDV was detected and remained detectable in the proboscises of all four insect species, and in the head/thorax portion of *Ae. aegypti*, *Cx. quinquefasciatus*, and *C. nubeculosus* for an extended length of time. For example, in insects which retained LSDV on their proboscis, the estimated duration of retention was 9 days for all four species, compared to 2.2 to 6.4 days in the body. This indicates that LSDV remains present for longer on the mouthparts of the insect than in the body and may therefore be available for transmission to a naive host for over a week.

Lengthy retention of poxviruses on insect mouthparts has been reported previously, with studies describing myxoma virus transmission by mosquitoes that had fed on lesions 25 and 29 days prior (6, 22), Shope fibroma virus transmission by mosquitoes that had fed on a lesions 35 days prior (23) and fowlpox virus transmission by mosquitoes that had fed on a lesions 41 days prior (4). In addition to lengthy retention times, other similarities between these poxviruses and LSDV include retention of the virus in or on mouthparts for longer than in other body parts, such as the abdomen (4, 22–24).

Another commonality among insect-borne poxviruses is the requirement for a cutaneous lesion in order for the insect to acquire virus (as shown in previous studies [4, 22] and this work). We therefore developed laboratory models of LSD skin lesions and used them to understand the impact of the cutaneous pathology on virus acquisition and retention. We found that feeding insects on blood spiked with LSDV at equivalent levels to those detected in the skin lesions enabled acquisition and retention comparable to that when feeding insects on LSD clinical animals or sections of skin nodules using an *ex vivo* model. This suggests that it is primarily a high quantity of LSDV that is required for acquisition and retention rather than, for example, a cofactor present in the cutaneous lesions. The high-titer laboratory model therefore represents a valuable tool for the study of LSDV in the vector, with the potential to reduce the use of animals in research.

Further work is now required to correlate the retention of LSDV on the proboscises and mouthparts of insects with their ability to initiate clinical disease in recipient cattle as the final step of the LSDV vector-borne transmission cycle, and to extrapolate from this knowledge to estimate how likely transmission will be in the field.

## MATERIALS AND METHODS

### Animal studies.

The animal study was performed in the high-containment animal facilities at The Pirbright Institute, as reported previously (20), under project license P2137C5BC from the UK Home Office according to the Animals (Scientific Procedures) Act of 1986, and it was approved by The Pirbright Institute Animal Welfare and Ethical Review Board. LSDV was propagated on Madin-Darby bovine kidney (MDBK) cells and semipurified through a 36% sucrose cushion prior to titration, as described previously (25). Five castrated Holstein-Friesian male cattle, aged between 127 and 140 days at challenge, were enrolled in the study. Four calves were challenged with 3 × 10^6^ PFU of LSDV intravenously and intradermally (20), while one remained unchallenged and was kept as an in-contact control. Cattle were monitored for 21 days post-challenge. One of the four inoculated calves developed clinical signs characteristic of systemic LSD, including fever, enlarged superficial lymph nodes, and multifocal cutaneous nodules (Fig. 1). The cutaneous nodules were first noted at 7 days postinoculation (dpi). This clinically affected calf was used as the *in vivo* source of LSDV for the insects in this study.

### *In vivo* insect feeding.

The four blood-feeding insect species used in the study were Aedes aegypti “Liverpool” strain, Culex quinquefasciatus TPRI line (Tropical Pesticides Research Institute, obtained from the London School of Hygiene and Tropical Medicine, London, United Kingdom), Stomoxys calcitrans (colony established in 2011 from individuals kindly provided by the Mosquito and Fly Research Unit, USDA Florida), and Culicoides nubeculosus (26). All insects were reared at The Pirbright Institute, as described previously (19). The age and sex composition of the insects at exposure was as follows: female *C. nubeculosus* between 0 and 2 days post-eclosion, female *Cx. quinquefasciatus* and *Ae. aegypti* at 5 to 7 days post-eclosion, and male and female *S. calcitrans* at an average of 4 days post-eclosion (range: 2 to 6 days). All adult insects were maintained on 10% sucrose and starved for 18 to 24 h before exposure to the calves. Feeding of the insects on the calf covered three different viremic periods: early viremia (9 and 10 dpi), peak viremia (11 and 12 dpi), and late viremia (18 to 20 dpi). During feeding, insects were held in a container covered with a mesh with apertures small enough to prevent escape yet allow feeding. The mesh was placed in close contact with the clipped or shaved skin of the calf. When required, normal skin adjacent to a cutaneous nodule was covered with adhesive tape to ensure that the feeding occurred on the lesioned skin. Two species were exposed to the donor on each day as follows: *C. nubeculosus* and *S. calcitrans* were exposed on 9, 11, and 18 dpi; and *Ae. aegypti* and *Cx. quinquefasciatus* were exposed on 10, 12, and 19 to 20 dpi. On each feeding occasion, the insect species were exposed either to a cutaneous nodule (lesion) or an area of skin without a nodule (normal skin). After feeding was complete, a sample of skin from each feeding site was collected using a microbiopsy technique (see below).

### *Ex vivo* insect feeding.

All four insect species were also fed on two *ex vivo* systems. The first was an artificial membrane system (Hemotek, United Kingdom) containing viremic blood collected from the clinically affected donor calf. The Hemotek was composed of a 3-mL metal reservoir holding the viremic blood and covered with a parafilm membrane which acted as a feeding surface for the insects. A heating unit ensured that the test blood remained at 37.4 to 38.0°C. Venous blood was collected from the jugular vein into tubes containing EDTA on 9 and 10 dpi (during early viremia) and on 11 and 12 dpi (peak viremia) before being loaded into the Hemotek reservoirs. The second *ex vivo* system incorporated skin lesions sourced from calves with clinical LSD into the Hemotek reservoirs. Samples of cutaneous nodules were collected postmortem from the calves with LSD and stored at −80°C. Thin slices of the cutaneous nodules were then generated using a DermaBlade Shave Biopsy Instrument (Verona, VA) and layered between two sheets of parafilm. This was then placed over a Hemotek containing defibrinated horse blood (TCS Biosciences Ltd.). Once feeding was complete, the skin was collected, cut into pieces, and homogenized in 1 mL high-glucose Dulbecco’s modified Eagle’s medium (DMEM) and LSDV genomic DNA was quantified.

### *In vitro* insect feeding.

*Ae. aegypti* were fed on LSDV-spiked horse blood using a Hemotek feeding unit. LSDV was propagated and semipurified as described above, then added to defibrinated horse blood (TCS Biosciences Ltd.) and used as a blood source in the Hemotek reservoir covered with a parafilm membrane. LSDV genomic DNA in the spiked blood was quantified using PCR (see below).

### Insect incubation.

Up to 2 h after exposure to a feed, source insects were anesthetized under CO_2_ and blood-engorged individuals (*Ae. aegypti*, *Cx. quinquefasciatus*, and *C. nubeculosus)* collected. Fed and non-fed *S. calcitrans* could not be distinguished by eye, therefore all *S. calcitrans* which had been exposed were processed. A subset of insects was frozen immediately (0 days post-feeding) following blood-feeding assessment under a microscope and stored at −80°C. The remaining blood-fed insects were maintained in darkness with a mean temperature of 24.1°C and relative humidity of 88.5% (monitored with RF513, Comark Instruments). After either 2, 4, or 8 dpf, surviving insects were collected and stored at −80°C. During their incubation period, all insects were maintained on 10% sucrose solution fed *ad libitum* and refreshed daily, except for *S. calcitrans* which were maintained with defibrinated horse blood (TCS Biosciences Ltd.) daily after 2 dpf.

### Insect dissection.

Insects were dissected under a light microscope over an ice-cold glass slide using sterile 25G 5/8” needles and surgical blades size no. 11. To prevent cross-contamination, a new microscope slide was used for each insect and needles/blades were changed after dissection of each body part. The legs were removed from *Ae. aegypti* and *Cx. quinquefasciatus* and the remainder dissected into three parts: proboscis, head-thorax (containing the salivary glands), and abdomen. *C. nubeculosus* were dissected into three parts (without leg removal): proboscis (including the most cranial third of the head), head-thorax (containing the salivary glands), and abdomen. This strategy aimed to isolate the salivary glands (in the head-thorax) from the abdomen. The salivary glands in *S. calcitrans* run into the abdomen (27), therefore, *S. calcitrans* legs were removed and then dissected into two parts: proboscis and head-thorax-abdomen. The dissected parts were digested overnight in 200 μL of tissue digest buffer (Tris-HCl [pH 8] 100 mM, NaCl 200 mM, SDS 0.2% [wt/vol], EDTA 5 mM) containing 2 μL of proteinase kinase (20 mg/mL, no. 100005393, Invitrogen) at 37°C. Following digestion, DNA was extracted using the whole volume of the digested product (200 μL) in a 96-well plate with the MagMAX CORE Nucleic Acid Purification kit (A32700; Applied Biosystems), using protocol A in a KingFisher Flex Magnetic Particle Processor (Applied Biosystems), and eluted in 50 μL of buffer.

### LSDV genome copy quantification.

One-mm skin biopsy samples were collected from each of the *in vivo* insect feeding areas on the clinical calf using a biopsy punch (Miltex). Skin was surgically prepared with alcoholic 2% clorhexidine wipes (Clinell) and anesthetized with either EMLA Cream 5% (lidocaine 25 mg/g, prilocaine 25 mg/g, Aspen Pharma Trading Ltd.) or Ametop Gel 4% (tetracaine hydrochloride 40 mg/g, Alliance Pharmaceuticals Ltd.). Skin biopsy samples were digested with 5 μL proteinase K in 95 μL PK buffer (448911, Applied Biosystems) for 30 min at 60°C, following the same extraction protocol described above. The *ex vivo* and *in vitro* feeding samples (blood in EDTA [200 μL], LSDV spiked defibrinated horse blood [100 μL], and homogenate of thin skin layers [100 μL]) did not require digestion and followed the same protocol for DNA extraction as described above, using the MagMAX CORE Nucleic Acid Purification kit.

LSDV genomic DNA was quantified by a PCR targeting the LSDV ORF068, adapted from the methods of Balinsky et al. (28), using the TaqMan Multiplex Master Mix (4461879; Life Technologies) or PathID (4388644; Life Technologies). A 20 μL reaction mixture was prepared using 5 μL of sample, 500 nM each primer (CaPV068F1 GGCGATGTCCATTCCCTG and CaPV068R1 AGCATTTCATTTCCGTGAGGA), 250 nM probe (CAATGGGTAAAAGATTTCTA), and nuclease-free water to the final volume. Samples were prepared in a 96-well plate and assayed using the Applied Biosystems 7500 Fast Real-Time PCR system with the following program conditions: 95°C for 20 sec and 45 cycles of 95°C for 15 s and 60°C for 60 s. For the quantification of LSDV, serial 1:10 dilutions of linear plasmid (GeneArt, Invitrogen) containing the entire ORF068 and ranging from 10 to 10^6^ copy numbers per reaction was used. Standard curves had an efficiency of >90%. LSDV genome copy numbers from samples with less than 10 or more than 10^6^ copy numbers per reaction were extrapolated from the linear regression curve.

### LSDV titration.

Eight-mm skin biopsy punches collected during the postmortem examination were placed in 1,000 μL DMEM (Life Technologies) supplemented with 5% fetal bovine serum (Antibody Production Services Ltd.), 100 IU/mL penicillin and 100 μg/mL streptomycin (Life Technologies), and 2.5 μg/mL amphotericin B (Life Technologies), and homogenized using Lysing Matrix A tubes (MP Biomedicals) in a BeadBug Microtube Homogenizer (Benchmark Scientific). After homogenization for 4 × 30 sec on 400 speed, the tissue was sonicated (Misonix 3000) for 2 × 30 s at power 4. The resulting suspension was transferred to a new tube and stored at −80°C prior to titration. Virus titration was carried out on MDBK cells as described previously (25).

### Statistical methods.

**Acquisition and retention of LSDV DNA by insects feeding on cattle.** Two analyses were carried out when exploring the differences in acquisition and retention among insect parts. In the first, all four species were included and differences in acquisition and retention between the proboscis and body were considered. For *Ae. aegypti*, *Cx. quinquefasciatus*, and *C. nubeculosus*, the body was considered positive if the head/thorax, abdomen, or both were positive for LSDV DNA. In the second analysis, only *Ae. aegypti*, *Cx. quinquefasciatus*, and *C. nubeculosus* were included and differences in acquisition and retention among the proboscis, head/thorax, and abdomen were considered.

In each analysis, two models were considered for the probability of an insect acquiring LSDV after feeding. The first depended on the virus source (normal skin, a lesion, or viremic blood from a clinical calf via a Hemotek), while the second depended on the level of viral DNA to which the insect was exposed (assumed to be given by the level in blood when feeding on normal skin or via a Hemotek or the level in the lesion when feeding on a lesion). In the first model, the probability of an insect acquiring LSDV after feeding is given by equation 1:

(1) $$\text{log}\left(\frac{p_{A}}{1-p_{A}}\right)=a_{0}^{\left(s\right)}+b_{j}^{\left(s\right)}+c_{k}^{\left(s\right)}$$

where *s* indicates the species, *j* is the virus source (normal skin, a lesion, or viremic blood from a clinical calf via a Hemotek), and *k* is the part of the insect tested. The coefficients, *a*_0_, *b*, and *c*, describe the intercept (i.e., baseline), the effect of virus sources and the effect of insect part on the probability of acquisition, respectively. Taking the exponential of the coefficients *b* or *c* [i.e., exp(*b*) or exp(*c*)] gives the odds ratio for comparing the effects of virus source and insect part on the probability of acquisition.

In the second model, the probability of acquisition is given by the equation

(2) $$\text{log}\left(\frac{p_{A}}{1-p_{A}}\right)=a_{0}^{\left(s\right)}+c_{k}^{\left(s\right)}+d^{\left(s\right)}V$$

where *s* indicates the species, *k* is the part of the insect tested, and *V* is the level of viral DNA to which the insect was exposed. The coefficients, *a*_0_, *c*, and *d*, indicate the intercept, the effect of insect part, and the dose-response on the probability of acquisition, respectively.

If an insect acquired LSDV, the probability of retaining it for *t* days post-feeding is given by equation 3

(3) $$p_{R}=\text{exp}\left(-\gamma_{k}^{\left(s\right)}t\right)$$

where γ is the species (*s*)- and insect part (*k*)-specific decay rate (so that the mean duration of retention is 1/γ). Consequently, the probability of an insect part being positive when tested is given by the equation

(4) $$p_{\text{POS}}=p_{A}p_{R}$$

where *p_A_* is given by equation 1 or equation 2 and pR is given by equation 3.

Species differences in the effect of virus source and insect part and of dose-response on acquisition and retention were incorporated by allowing these parameters to vary among species through hierarchical structure in the parameters, so that

(5) $$\begin{array}{l} a_{0}^{\left(s\right)}\sim \text{Normal}(\mu_{a},\sigma_{a})\\ b_{j}^{\left(s\right)}\sim \text{Normal}(\mu_{b_{j}},\sigma_{b_{j}})\\ c_{k}^{\left(s\right)}\sim \text{Normal}(\mu_{c_{k}},\sigma_{c_{k}})\\ d^{\left(s\right)}\sim \text{Normal}(\mu_{d},\sigma_{d})\\ \gamma_{k}^{\left(s\right)}\sim \text{Gamma}(a_{\gamma_{k}},b_{\gamma_{k}}) \end{array}$$

where *μ* and *σ* are the mean and standard deviation for the normal distribution and *a* and *b* are the shape and scale parameters for the gamma distribution.

Parameters in the models were estimated in a Bayesian framework. The likelihood for the data is given by

(6) $$L=\prod_{i}{\left(p_{\text{POS}}^{\left(i\right)}\right)}^{\delta_{i}}{\left(1-p_{\text{POS}}^{\left(i\right)}\right)}^{1-\delta_{i}}$$

where *δ_i_* is a variable indicating whether insect *i* was positive (*δ_i_* = 1) or negative (*δ_i_* = 0) for LSDV DNA when tested. Hierarchical priors were used for species-specific parameters, with non-informative priors used for the hierarchical distribution parameters: normal with mean 0 and standard deviation 10 for the *μ*s and exponential with mean 100 for the *σ*s, *a*s, and *b*s.

The methods were implemented using OpenBUGS (version 3.2.3; https://www.mrc-bsu.cam.ac.uk/software/bugs/openbugs/). Two chains of 120,000 iterations each were generated, with the first 20,000 iterations discarded to allow for burn-in of the chains. Chains were subsequently thinned by selecting every 20th sample to reduce autocorrelation of the samples. Convergence of the chains was monitored visually and using the Gelman-Rubin statistic in OpenBUGS.

Different models for variation in acquisition and retention among species were compared using the deviance information criterion (DIC) (29). The two models for the probability of acquisition of LSDV were compared using posterior predictive *P* values. Specifically, the joint posterior distribution for the model was sampled and the probability that each insect part was positive for viral DNA computed. Whether or not the insect was positive when tested was then simulated, and the observed outcomes were compared to the simulated ones. This procedure was repeated multiple times, and the proportion of samples for which the observed and simulated outcomes matched was computed (i.e., the posterior predictive *P* value).

**Acquisition and retention of LSDV DNA by insects feeding *ex vivo*.** A similar approach to that described above was used when assessing the acquisition and retention of LSDV DNA by *Ae. aegypti* after feeding *ex vivo*. In this case, the probability of an insect acquiring LSDV after feeding *ex vivo* is given by

(7) $$\text{log}\left(\frac{p_{A}}{1-p_{A}}\right)=a+b_{j}+c_{k}$$

where *j* indicates the source of virus (from a lesion *ex vivo*, blood/virus mix at high titer, or blood/virus mix at low titer) and *k* indicates the part of the insect tested (proboscis, head/thorax, or abdomen). The coefficients *a*, *b*, and *c* describe the intercept (i.e., baseline), the effect of virus source, and the effect of insect part on the probability of acquisition, respectively. The probability of retention is given by equation 3, the probability of an insect part being positive by equation 4, and the likelihood for the data by equation 6.

A second analysis was carried out in which the data for *Ae. aegypti* from the animal experiment and the *ex vivo* study were combined so that there were six sources of virus (from a lesion *ex vivo*, blood/virus mix at high titer, blood/virus mix at low titer, a lesion on a clinical calf, normal skin on a clinical calf, or viremic blood from a clinical calf).

The methods were implemented using OpenBUGS (version 3.2.3). Two chains of 150,000 iterations each were generated, with the first 50,000 iterations discarded to allow for burn-in of the chains. Chains were subsequently thinned by selecting every 20th sample to reduce autocorrelation of the samples. Convergence of the chains was monitored visually and using the Gelman-Rubin statistic in OpenBUGS. Different models for variation in acquisition and retention were compared using the deviance information criterion.

**Levels of LSDV DNA retained after insect feeding *ex vivo*.** The level of viral DNA retained on different insect parts and how this depended on the source of virus and days post-feeding was assessed using a linear model. More specifically, the response variable was log_10_ copy number, with insect part, source of virus, and days post-feeding as explanatory variables. Model selection proceeded by stepwise deletion of nonsignificant (*P* > 0.05) terms (as judged by *F*-tests), starting from a model including all three explanatory variables and interactions between them. Differences among factors in the final model were explored using Tukey’s multiple-comparison tests. The analysis was implemented in R (version 4.0.2). Because of the small number of positive samples from insects feeding on blood/virus mix at low titer, these were excluded from the analysis.

### Data availability.

The data and code for the analyses presented in this paper are available online (30).

## APPENDIX

**COMPARISON OF MODELS FOR THE ACQUISITION AND RETENTION OF LUMPY SKIN DISEASE VIRUS BY BLOOD-FEEDING INSECTS**

In this appendix, we provide the full results comparing different models for the acquisition and retention of LSDV by insects after feeding on an infected bovine (Table A1) and for the acquisition and retention of LSDV by *Ae. aegypti* after feeding *ex vivo* (Table A2).

**TABLE A1:** Comparison of different models assessing variation in probability of acquisition and retention of lumpy skin disease virus among insect species^*a*^

Acquisition		Retention	DIC^*b*^	
Virus source	Insect part	Insect part	4 spp.	3 spp.
Y,S	Y,S	Y,S	**474.6**	**478.3**
Y	Y,S	Y,S	483.5	500.9
Y,S	Y	Y,S	479.6	480.9
Y	Y	Y,S	491.4	502.5
N	Y,S	Y,S	656.0	647.3
Y,S	N	Y,S	578.4	593.7
Y,S	Y,S	Y	481.7	480.9
Y,S	Y,S	S	487.4	487.4

a Y,S: varies by factor (i.e., virus source or insect part) and species; Y: varies by factor, but not species; S: varies by species, but not factor; N: does not vary by factor or species.

b A model with a smaller DIC (deviance information criterion) is preferred to one with a higher DIC; the model with its DIC shown in bold is the preferred one.

**TABLE A2:** Comparison of different models assessing variation in probability of acquisition and retention of lumpy skin disease virus in Aedes aegypti after feeding *ex vivo*^*a*^

Acquisition		Retention	DIC^*b*^	
Virus source	Insect part	Insect part	*Ex vivo* study only	*Ex vivo* and animal studies
Y	N	Y	606.3	857.2
N	Y	Y	590.2	928.4
Y	Y	N	550.4	758.3
Y	Y	Y	**530.2**	**730.7**

a Y: varies by factor; N: does not vary by factor.

b A model with a smaller DIC (deviance information criterion) is preferred to one with a higher DIC; the model with its DIC shown in bold is the one preferred.
